# Acceptability and user experiences of a patient-held smart card for antenatal services in Nigeria: a qualitative study

**DOI:** 10.1186/s12884-023-05494-9

**Published:** 2023-03-22

**Authors:** Ijeoma Uchenna Itanyi, Juliet Iwelunmor, John Olajide Olawepo, Semiu Gbadamosi, Alexandra Ezeonu, Adaeze Okoli, Amaka Grace Ogidi, Donaldson Conserve, Byron Powell, Chima Ariel Onoka, Echezona Edozie Ezeanolue

**Affiliations:** 1 Department of Community Medicine, College of Medicine, University of Nigeria Nsukka, Enugu, Nigeria; 2 Center for Translation and Implementation Research, University of Nigeria Nsukka, Enugu, Nigeria; 3 Department of Behavioral Science and Health Education, Saint Louis University, Saint Louis, USA; 4 Department of Health Sciences, Bouvé College of Health Sciences, Northeastern University, Boston, MA USA; 5 Robert Stempel College of Public Health & Social Work, Florida International University, Miami, FL USA; 6 Department of Prevention and Community Health, Milken Institute School of Public Health, George Washington University, Washington, USA; 7 Brown School, Washington University in St. Louis, Washington, USA

**Keywords:** Acceptability, User perception, mHealth, Smart cards, Mobile app, Maternal health, Antenatal services, Implementation research, Nigeria, Resource-limited setting

## Abstract

**Background:**

Poor maternal, newborn and child health outcomes remain a major public health challenge in Nigeria. Mobile health (mHealth) interventions such as patient-held smart cards have been proposed as effective solutions to improve maternal health outcomes. Our objectives were to assess the acceptability and experiences of pregnant women with the use of a patient-held smartcard for antenatal services in Nigeria.

**Methods:**

Using focus group discussions, qualitative data were obtained from 35 pregnant women attending antenatal services in four Local Government Areas (LGAs) in Benue State, Nigeria. The audio-recorded data were transcribed and analyzed using framework analysis techniques such as the PEN-3 cultural model as a guide.

**Results:**

The participants were 18–44 years of age (median age: 24 years), all were married and the majority were farmers. Most of the participants had accepted and used the smartcards for antenatal services. The most common positive perceptions about the smartcards were their ability to be used across multiple health facilities, the preference for storage of the women’s medical information on the smartcards compared to the usual paper-based system, and shorter waiting times at the clinics. Notable facilitators to using the smartcards were its provision at the “Baby showers” which were already acceptable to the women, access to free medical screenings, and ease of storage and retrieval of health records from the cards. Costs associated with health services was reported as a major barrier to using the smartcards. Support from health workers, program staff and family members, particularly spouses, encouraged the participants to use the smartcards.

**Conclusion:**

These findings revealed that patient-held smart card for maternal health care services is acceptable by women utilizing antenatal services in Nigeria. Understanding perceptions, barriers, facilitators, and supportive systems that enhance the use of these smart cards may facilitate the development of lifesaving mobile health platforms that have the potential to achieve antenatal, delivery, and postnatal targets in a resource-limited setting.

## Introduction

A critical unanswered question in many low and middle-income countries is how to identify pregnant women at risk of infections and diseases early and implement interventions to improve infants’ birth outcomes and survival [1]. This is because there are often delays in implementing life-saving interventions due to difficulties in accessing care and problems with quality of care provided [2–5]. Information gaps on maternal and child health interventions as well as structural barriers and behavioral limitations on the demand side hinder access to life-saving interventions [4, 6–8]. These challenges contribute to low and delayed uptake of maternal and child health interventions in low-income countries. As a result, implementation of simple, cost-effective, culturally adapted and sustainable interventions are needed to save more mothers’ and children’s lives [4].

Poor maternal, newborn and child health (MNCH) outcomes are a major public health challenge in Nigeria, where an estimated 10% of all global under-five and maternal deaths – more than one million newborn, infant and child deaths, and more than 50,000 maternal deaths - occur every year [9]. Despite improvements overtime, systematic reviews suggest that Nigeria lags behind on global and sub-Saharan Africa averages on maternal and child health indicators since documentation of national MNCH statistics began in the early 1990s [10–14]. Innovative strategies are needed to improve health coverage and reduce poor MNCH outcomes in Nigeria [8, 11]. Mobile health (mHealth), defined as mobile communication technologies to support health care [15, 16], is one potential strategy that may improve MNCH outcomes in Nigeria. Nigeria is Africa’s largest mobile market with over 150 million mobile phone-users and a high penetration of internet services through mobile networks [17]. With the continuous growth of mobile network and penetration of mobile devices in developing countries such as Nigeria, mHealth interventions with integrated data and medical decision algorithm provide innovative opportunities to address health priorities [16, 18–21] and enhance the implementation of evidence-based interventions to improve maternal and child health outcomes in Nigeria [22]. These interventions also have the capacity to reduce human error, expedite tasks, and can provide researchers and program managers with the tools needed to address challenges that thwart the progress of MNCH programs in Nigeria. These include increasing access to health information [23], reducing turnaround times for receipt of laboratory test results [24], improving decision-making, and ultimately saving lives [25]. Among patients, mHealth interventions have the potential to empower them with the education and knowledge needed to make better health-related decisions, adhere to medical regimen, and have better control of their own health outcomes [16, 26–31].

Our team developed a pilot intervention to assess the feasibility of an integrated mobile health platform to improve maternal health outcomes in resource-poor settings. This mobile health platform is able to collect data from community-based programs, embed the collected data into a smart card, and read the smart card using a mobile phone-based app in the health facility, without the need of internet access [1, 22]. A detailed description of the patient-health smartcard has been published previously [22].

In this paper, we report on the acceptability and user experiences of pregnant women who participated in this pilot smart card mHealth intervention in Nigeria, and the role of culture in shaping the health beliefs and health behaviours of these women in accepting and using the mHealth intervention. Understanding the perspectives of these women and the potential facilitators and barriers to accepting and using this mHealth intervention, will ultimately inform the development of implementation strategies to improve uptake of mobile health interventions in resource-poor settings.

We define acceptability as the extent to which an innovation is attractive, agreeable, or palatable, based on the taxonomy of implementation outcomes used in implementation research [32]. Evaluating the acceptability of mobile health technologies for MNCH services among key stakeholders in Nigeria, particularly pregnant women utilizing these services is important for several reasons. First, mobile health technologies are complex in nature and can consist of several interacting components [33]. They may also be delivered at different levels (patients, healthcare workers), with individuals with varying degree of literacy, and in different settings such as in community or health care settings. Successful implementation depends on the acceptability of mobile health technologies to both recipients (i.e., patients) and intervention deliverers (i.e., researchers or healthcare professionals) [34]. Secondly, the content, context, and quality of services received using mobile health technologies may all have implications for acceptability [33, 34]. If a mobile health technology for MNCH is considered acceptable, patients are more likely to adhere to recommendations provided and benefit from improved clinical outcomes. Likewise, from the perspective of healthcare professionals, if an intervention that uses mobile health technology is considered to have low acceptability, the intervention may not be delivered to recipients as intended by the intervention developers, which in turn may impact the overall effectiveness of the intervention.

## Methods

### Study context and participant recruitment

Pregnant women residing in four local government areas (i.e., counties) in Benue State, Nigeria, were invited to participate in this study. Participants were part of the pilot smart card intervention in Benue State, Nigeria. Maximum variation purposive sampling approach was used to recruit participants of varying socio-demographic backgrounds (e.g., age, occupation, education, income, urban vs. rural residence, primigravidae vs. multiparous women, etc.) from their communities in these four counties. Recruitment continued until saturation was achieved during data collection.

### Study design

Data for this study were derived from focus group discussions (FGDs) held with participants in January and May, 2017. Focus group discussions were appropriate to explore the perceived barriers and/or facilitators that may influence use of mobile health technologies for MNCH services [35–37]. Further, focus groups provide the opportunity to identify lived experiences not captured in quantitative research and to probe deeply on comments made by group members [35–37]. All the women who were approached to participate agreed and provided written and verbal consent. Biodata was obtained from all participants. A public health professional (study co-investigator) trained in qualitative research methods, led the FGDs as the moderator with a research assistant trained in notetaking.

### Theoretical framework

This study was guided by the PEN-3 cultural model [38–40] which centralizes culture in the study of health beliefs, behaviors, and health outcomes. The PEN-3 cultural model was used as the theoretical framework to identify the perceptions, enablers, and nurturers related to the use of a mobile health technology for MNCH services. The PEN-3 cultural model consists of three primary domains: Cultural Identity, Relationships and Expectations, and Cultural Empowerment. Each domain comprises three factors that form the acronym PEN: Person, Extended Family, and Neighborhood (Cultural Identity domain); Perceptions, Enablers, and Nurturers (Relationships and Expectation domain); Positive, Existential and Negative (Cultural Empowerment domain). [39] Particularly, we used the relationships and expectations domain to explore perceptions about the smart card, the societal resources that promote or discourage use of the smart cards, and the influence of family and kin in nurturing decisions about use of the smart cards. We also used the cultural empowerment domain to investigate cultural beliefs and practices that are positive, cultural values and beliefs that are existential and have no harmful health consequences, and negative cultural beliefs or practices that affect utilization of the smart cards. The focus group discussions lasted between 60 and 75 min and discussions were audio-recorded with permission from participants. To preserve confidentiality during discussions, names or other identifiers were not used. The study was approved by the Institutional Review Board of the University of Nevada, Reno, and the Nigerian National Health Research Ethics Committee.

### Data analysis

All focus group discussion recordings were transcribed *verbatim* and analyzed thematically using framework analysis approach. Framework analysis is a systematic process for sifting, charting and sorting material according to key issues and themes [36]. This process involves data familiarization; identifying a thematic framework; indexing; charting; and mapping and interpretation [35–37, 41]. The research team began by reading the transcripts and field notes in its entirety to familiarize ourselves with the data. After reading the transcripts, four members of the research team collaboratively developed a coding frame. The coding frame was developed deductively by writing down recurring concepts and themes, while using the PEN-3 cultural model as a guide. Next, a list of open codes was sorted, relabeled and condensed into a smaller number of broad themes. This was followed by creating closed coding where codes related to the same theme were grouped together. Further, final codes and themes were discussed among the research team to reach a consensus. Two members of the research team independently applied the coding frame to the first transcript. The full research team then reviewed, discussed and resolved any inconsistencies before the coding of the rest of the transcripts. The main emerging themes were related to perceptions of mHealth, enablers for mHealth use and nurturers of mHealth use. These themes were further explored through a thorough review of each transcript. The final analysis framework was created to distinguish between positive, existential, and negative attributes related to the emerged themes – perceptions, enablers, and nurturers. The FGDs were conducted and analysed by members of the research team who have qualitative research skills. The processes were led by 2 members of the research team, one a physician with a Ph.D focused on health systems and qualitative research (CAO), the other holds a Ph.D in qualitative research and co-developed the PEN-3 cultural model (JI).

## Results

### Characteristics of the participants

A total of 35 women between ages 18 and 44 years (median age = 24 years), participated in the study. All the participants were married women and 89% (n = 31) were farmers. Most of the participants (n = 33) had some level of education. Most of the participants spoke two languages (Tiv and English). Majority of the participants (n = 31) made ₦20,000 (twenty thousand Nigerian Naira) or less per month. (Table 1)

**Table 1:** Characteristics of female participants of focus group discussions in Benue State, Nigeria

Characteristic (N = 35)	Frequency
**LGA**	
Buruku	3
Gwer West	11
Konshisha	9
Tarka	12
**Number of children**	
None	6
1 or 2	16
>2	12
Missing	1
**Level of education**	
None	2
Completed primary	7
Completed junior secondary	10
Completed senior secondary	11
Some post-secondary	3
Completed post-secondary	2
**Main language spoken**	
English	21
Tiv	14
**Income**	
₦0 – ₦20,000	31
₦20,001 - ₦50,000	3
>₦50,000	1

All the participants reported that they had received their mHealth cards at least a month prior to the discussions about its use. All the women reported that they received consent from their spouses before participating in the FGDs. 22 of the women reported that they had used the smartcard, which was scanned at the hospital with a phone to display their information. The reasons reported for not using the smartcard included not knowing how to use the card, not knowing the card can be used at other health facilities, forgetting to go to the hospital with the cards, not having had an appointment since given the smartcard, and not having gone to the clinic personally. One participant reported that she stopped using the smart card after her husband took the card to the hospital without her permission. The participants’ responses are summarized in Table 2.

**Table 2:** PEN-3 table of analysis showing findings in each domain

Cultural Empowerment				
Relationship/ Expectation	Domains	Positive	Existential	Negative
	Perceptions	-Perceived the card to be beneficial. -Understood the purpose of the card.	-Not sure of the scope of information in the card. -Not certain why the card is free. -Not sure if the smart card program will apply to their babies.	-Thoughts that the card gives access to free medical screening. -Did not understand why they were selected. -Afraid they received the card because of an illness.
	Enablers	-The benefits that they received by using the card: card reduced the time spent at the facility, ease of utilizing services available at the clinic, reduced cost by not repeating laboratory investigations that have results stored on the cards. -Demand for use of the card by healthcare worker. -Availability of LGA Research Assistants and healthcare workers to give information and clarification. -Card can be used in any of the participating facilities. -The positive experience from the “Baby Shower” program	-No cultural belief against the use of the cards. -Not having had a hospital appointment since given the card.	-Cost of treatment at the health facilities. -Use of the card by someone other than client. -Not knowing how to use the card. -Not knowing the card can be used at other participating health facilities. -Forgetting to go to the hospital with the cards.
	Nurturers	-Approval from husbands -Support from household members	-Family accepts use of orthodox medicine/healthcare	-Negative ideas from families and neighbours

### Perceptions (attitudes towards mHealth card and its use)

#### Positive perceptions

Most of the participants understood the purpose of the card. Almost all the participants perceived the card to be important for their healthcare and likened it to an ATM (Automated Teller Machine) card that contained all their biodata and investigation results. They believed the card was made for their health benefit as it reduced their healthcare cost as they did not have to repeat some laboratory investigations. All participants who had used the card were happy that they did not have to spend money to repeat laboratory investigations or have additional needle pricks. Some of them were happy that it could be used even when they are away from home. Most of the women acknowledged that the mHealth card made it faster and easier for the healthcare workers at the hospitals to retrieve their laboratory results stored on their cards instead of searching for hospital folders, resulting in shorter waiting times. Most of them reported that the card helped them know more about their health status and helped the healthcare worker to know their diagnosis, hence, reducing the time spent at the facility as the users were asked fewer questions at the health facilities.

*“I like using the card at the hospital, it saves the work of checking files or folders. It also produces my results faster.” (Participant 19)*.

When describing the benefits associated with using the card, some of the participants expressed that they liked the ability to use the mHealth card in different health facilities. This would allow for easy transfer of information from one health facility to another. In this regard, majority of the participants viewed the mHealth card to be compatible and usable in various health facilities in their community.

*“I feel if I go to a different hospital, I can still use the card. So, I like it. Also, the card has helped me know more about myself as regards my health…It contains my health information, so I like it.” (Participant 15)*.

Negative perceptions Some of the participants did not understand why they were selected to receive the cards and were concerned at the first instance. Some participants were initially afraid that they received the card because of an illness. One participant felt it could be due to issues with her pregnancy while another felt she may have a health challenge; however, they got convinced that presenting the card at the hospital will make their treatment easier. Some other participants did not have accurate information about the cards. Some were not certain why the card was free while others were not sure if the program will apply to their babies. Some felt they will incur additional costs by taking the card to the hospital while some others felt the card will give them access to free medical services. Another group of participants reported that they did not think they could use the mHealth cards in other health facilities serving pregnant women. Most of the participants were not sure if the card captures every data about their health or only the laboratory investigations done during the “Baby Shower” program. The “Baby Shower” program was a health intervention delivered in churches to celebrate pregnancy and deliver integrated onsite health screening for pregnant women.[42].

*“We were many that participated in the baby-shower program why is the cards given to only few of us? I became worried when I noticed this”*. *(Participant 8)*

*“People should be told clearly that if they are using this card, their treatment will be free. I felt I will be charged for taking the card to the hospital.” (Participant 6a)*.

### Enablers (factors that represent barriers to or facilitators of mHealth card use)

#### Positive enablers

When describing the facilitators that contributed to their use of the cards, participants who had used the mHealth cards stated that the healthcare worker’s request for the card in the facilities they visited made them use the cards. Most participants reported they had been asked for the card at the health facility. The fact that the cards could be used in any of the participating facilities also enabled more women to use the cards. Again, the availability of LGA Research Assistants and healthcare workers to give information and clarification aided the use of the cards. Participants discussed how receiving assistance in the form of technical support and response to questions from the program facilitators, the nurses and even the doctors encouraged their use of the mHealth cards. One participant shared that the on-going discussions about the card with program facilitators were important in supporting her use of the card. When describing how to encourage participants to use the card, it was noted that the women should be provided with more information on the card.

*“Yes, the facility workers were always asking me about the card whenever I went for ANC visits. At the facility, they asked me to always bring the card, they always scan it and return to me.” (Participant 2a)*.

*“Whenever I have questions about the card, I asked the program facilitator, the nurse or doctor. They give me more information concerning the card.” (Participant 5b)*.

The positive experience from the “Baby Shower” program was another enabler. Some participants perceived the mHealth card to be beneficial by linking it to the incentives received during the “Baby Shower” program. They liked that the card was free and came with some form of support. They were thankful to and encouraged the sponsors of the “Baby Shower” program.

*“I liked the gifts given to me during the baby shower so when the card was given, I knew it was another good thing so when I was invited over for the interview, I didn’t hesitate.” (Participant 18)*.

The participants were unanimous in their response that their cultural beliefs will not hinder their proper use of the cards.

#### Negative enablers

When describing factors that acted as barriers to using the cards, the costs associated with seeking treatment was cited as the most prevalent barrier. Some participants believed that although the cards allowed them to have various tests for free, subsequent costs associated with treatment were among their reasons for not using the cards. One participant shared the following:

*“Tests were done and we have our results, just that there has been no money at the moment to go to the hospital for treatment.” (Participant 4b)*.

Other barriers reported by the women include inadequate information on the use of the card and unauthorized use of the card. A few women reported that poor knowledge of how to use the card and not knowing that the card could be used at other participating health facilities prevented them from using the cards. One participant reported an unauthorized use of the card by her husband at a health facility. This unauthorised use was a barrier to her further use of the card.

*“Even though I accept the card and know that is for my health interest, I did not use the card initially because I thought the card cannot be used at the hospital where I attend antenatal care”. (Participant 10)*

*“I have always forgotten to take my card to the hospital”…….bursts into laughter. (Participant 17)*

### Nurturers (the role of support systems with using the m-health card)

#### Positive nurturers

The influence of family and neighbours on the acceptability and use of the mHealth cards were highlighted. All the participants reported that they received consent and support from their spouses to use the card. Most participants also reported that they received support from members of their households. When participants were asked to describe key individuals who supported their use of the mHealth cards, participants said that receiving support from their family members, particularly their spouse increased their use of the cards.

*“I only showed the card to my husband and he accepted that I keep and make use of the card.” (Participant 7a)*.

*“When I showed it to my household, I did not get any negative comments or rejection of the card.” (Participant 4a)*.

#### Negative nurturers

A few of the participants reported that they were discouraged from using the cards by friends, neighbours and relatives. A few of the women were mocked that the card will not have a lasting impact given that similar projects have failed in the past. Others were told they received the card because they were sick.

*“I was told by people around me that the card is given to those with HIV/AIDS only and this prompted me to take it to the hospital for verification which I found out it was not true of what people said.” (Participant 14)*.

*“Some people that I showed the card said negative things about the card, that the project will not achieve it aims and objectives since other organisations had come with similar projects that never stayed to the end”*. *(Participant 5a)*

## Discussion

This study described the perceptions, enablers (barriers and facilitators) and nurturers to the acceptability and use of an mHealth smart card by pregnant women for MNCH services. As the perceptions of pregnant women and their use of mobile health services in Nigeria have not been fully documented, the findings on their perceptions, enablers, and nurturers contribute to the literature on the role mobile health platforms can play in improving MNCH services in the country. The present study revealed different perceptions of the use of the mHealth cards, the resources that enable or act as barriers towards use and the role of support systems in encouraging participants’ use of the cards.

The participants expressed different views about the benefits of the mHealth cards. For instance, they found the provision of their medical information on the card as an acceptable and potentially preferred method for keeping their medical records instead of a paper-based folder or card system. Related mHealth studies in Kenya and Ethiopia, found a preference for mHealth technologies, especially among women utilizing antenatal care services. In Kenya, Bastelaer and colleagues (2015) found high levels of uptake, with participants identifying several benefits with using a mHealth platform for maternal health care services [43]. In Ethiopia, Shiferaw and colleagues (2016) found that using a mHealth platform that includes decision support, sending reminders about subsequent visits and educational messages, significantly increased utilization of delivery and postnatal care services [44]. Mobile health platforms are feasible and could be viable options for storing maternal medical information, including laboratory data at the point of delivery that could ultimately enhance the implementation of proven evidence-based MNCH services in resource-constrained settings.

In addition to the perceptions, we identified factors that facilitated or hindered use of the mHealth cards (positive and negative enablers respectively). The participants acknowledged that the healthcare workers and the research assistants facilitated their use of the mHealth cards. The healthcare workers demanded for the mHealth cards at the facility providing an opportunity for more communication between the healthcare workers and the patients. A systematic review of utilization of mHealth by health workers substantiated that mHealth facilitates communication between health workers and patients. [45] The availability of the healthcare workers and the research assistants to give more information about the card also increased demand to use the cards. A similar finding was reported in a study of facilitators and barriers to use of mobile health applications in low-income populations where the participants relied on community care workers for clarifications. [46]

One of the main issues raised by participants in this study was potential costs associated with using the cards. However, some participants recommended consistent messaging focused on discussing the benefits of using the cards, particularly for the free health tests and the “Baby Shower” incentives, as it may drive more participants to become engaged with the mHealth platform in general. The finding that some of the women did not understand how to use the card highlights the need for more comprehensive education on the benefits and processes for use of mHealth technologies in other resource-limited settings. The finding about unauthorized use of the smart card by a participant’s spouse highlights the importance of measures to ensure confidentiality and privacy with mHealth interventions. In this study, the health workers were trained to ask some security questions to a client before releasing any health information. While this may prevent unauthorized use of the smart card, additional security features like facial recognition have a higher potential to protect patients’ privacy and confidentiality.

This study identified spouses and other family members as supportive systems that encouraged participants to use their mHealth cards. This finding highlights the vital role that spouses and a strong family support system can play in the use of mHealth technologies for MNCH services. The family support system remains integral to dispelling of myths and negative ideas from friends and other individuals in the women’s network. For further advances in the use of mHealth technologies, we posit that family members and community health workers can play an important role in engaging women to increase use of mHealth technologies for MNCH care utilization.

Taken together, these findings suggest that investment in marginally low-cost, broadly accessible mobile health systems can serve as vehicles for maternal health care services in Nigeria. The continuous connectivity between individuals and mHealth platforms creates opportunities for improving MNCH services in resource-limited settings.

### Limitations

There are limitations to this study worth mentioning. First, the relatively small sample size and recruitment of participants from one geographical area in Nigeria, with majority reporting low educational attainment suggests that these results may not be generalizable to other women utilizing smart cards for MNCH services in other parts of Nigeria or women with higher educational attainment. Second, since the data was collected during focus group discussions, there is possibility that the information provided by participants may be influenced by social desirability bias. Additionally, qualitative data are subject to researcher bias, which we sought to address by having the focus group discussions conducted by researchers with extensive qualitative research experience. Finally, while participants found the smart cards to be acceptable, we did not speak to the health care providers, which limits our insights into healthcare providers’ attitudes and perceptions on the acceptability and use of the smart cards. Further research in this area could explore health care providers’ perceptions about smart cards, the resources that may act as barriers or facilitate their use of these smart cards, and the role leaders and supportive systems could play to further enhance their ability to provide accurate information on the benefits of the cards to women utilizing MNCH services. We also recommend a systematic review of implementation strategies to increase acceptability and adoption of mhealth interventions for maternal health in low- and middle-income countries.

## Conclusion

In conclusion, this qualitative investigation suggests that a patient-held smart card for maternal health care services is acceptable to women utilizing MNCH services in Nigeria. Understanding perceptions, barriers, facilitators, and supportive systems that enhance the use of these smart cards may facilitate the development of lifesaving mHealth interventions that have the potential to achieve MNCH targets in resource-limited settings, and inform the development of implementation strategies to improve uptake of mobile health interventions in such settings.

## Data Availability

The datasets analyzed during the current study are not publicly available due to the vulnerable nature of the participants (pregnant women) but are available from the corresponding author on reasonable request.

## Abbreviations

AIDS: Acquired immunodeficiency syndrome
ANC: Antenatal care
FGDs: Focus group discussions
HIV: Human immunodeficiency virus
LGA: Local government area
MNCH: Maternal, newborn and child health
